# Health promotion in school environment in Brazil

**DOI:** 10.1590/S1518-8787.2017051006709

**Published:** 2017-03-31

**Authors:** Rogério Lessa Horta, Cristine Scattolin Andersen, Raquel Oliveira Pinto, Bernardo Lessa Horta, Maryane Oliveira-Campos, Marco Antonio Ratzsch de Andreazzi, Deborah Carvalho Malta

**Affiliations:** I Programa de Pós-Graduação em Saúde Coletiva. Unidade de Pesquisa e Pós-Graduação. Universidade do Vale do Rio dos Sinos. São Leopoldo, RS, Brasil; II Núcleo de Saúde e Segurança do Trabalho. Gestão de Pessoas. Instituto Federal de Educação, Ciência e Tecnologia. Farroupilha, RS, Brasil; III Hospital Escola. Universidade Federal de Pelotas. Empresa Brasileira de Serviços Hospitalares. Pelotas, RS, Brasil; IV Programa de Pós-Graduação em Epidemiologia. Faculdade de Medicina. Universidade Federal de Pelotas. Pelotas, RS, Brasil; V Departamento de Ciências Básicas e da Saúde. Universidade Federal dos Vales do Jequitinhonha e Mucuri. Diamantina, MG, Brasil; VI Coordenação de População e Indicadores Sociais. Diretoria de Pesquisa. Instituto Brasileiro de Geografia e Estatística. Rio de Janeiro, RJ, Brasil; VII Departamento de Vigilância de Doenças e Agravos Não Transmissíveis e Promoção da Saúde. Secretaria de Vigilância em Saúde. Ministério da Saúde. Brasília, DF, Brasil; VIII Departamento de Enfermagem Materno Infantil e Saúde Pública. Escola de Enfermagem. Universidade Federal de Minas Gerais. Belo Horizonte, MG, Brasil

**Keywords:** Adolescent, School Health, Health Promotion, Socioeconomic Factors, Health Inequalities, Social Inequity

## Abstract

**OBJECTIVE:**

Evaluate the school environments to which ninth-year students are exposed in Brazil and in the five regions of the country according to health promotion guidelines.

**METHODS:**

Cross-sectional study from 2012, with a representative sample of Brazil and its macroregions. We interviewed ninth-year schoolchildren and managers of public and private schools. We proposed a score of health promotion in the school environment (EPSAE) and estimated the distribution of school members according to this score. Crude and adjusted odds ratios (OR) were used, by ordinal regression, to determine the schoolchildren and schools with higher scores, according to the independent variables.

**RESULTS:**

A student is more likely to attend a school with a higher EPSAE in the South (OR = 2.80; 95%CI 2.67–2.93) if the school is private (OR = 4.52; 95%CI 4.25–4.81) and located in a state capital, as well as if the student is 15 years of age or older, has a paid job, or has parents with higher education.

**CONCLUSIONS:**

The inequalities among the country’s regions and schools are significant, demonstrating the need for resources and actions that promote greater equity.

## RESUMO

**OBJETIVO:**

Avaliar os ambientes escolares aos quais estão expostos estudantes do nono ano no Brasil e nas cinco regiões do país segundo diretrizes de promoção da saúde.

**MÉTODOS:**

Estudo transversal, de 2012, com amostra representativa do Brasil e suas macrorregiões. Escolares do nono ano e gestores de escolas públicas e privadas foram entrevistados. Foi proposto o Escore de Promoção de Saúde no Ambiente Escolar (EPSAE) e foi estimada a distribuição dos escolares segundo esse escore e segundo *odds ratio* (OR) brutas e ajustadas, por regressão ordinal, para exposição dos escolares a escolas com escores mais elevados, segundo as variáveis independentes.

**RESULTADOS:**

Um escolar tem mais probabilidade de frequentar escola com EPSAE elevado na região Sul (OR = 2,80; IC95% 2,67–2,93) se a escola for privada privada (OR = 4,52; IC95% 4,25–4,81) e estiver localizada em capital de estado e se o escolar tiver 15 anos de idade ou mais, exercer atividade remunerada ou tiver mãe ou pai com escolaridade superior.

**CONCLUSÕES:**

São importantes as desigualdades entre regiões do país e entre redes de ensino, tornando clara a necessidade de alocação de recursos e ações que promovam maior equidade.

## INTRODUCTION

Health and school are mutually interconnected. Health indicators improve with education, and good health improves school performance^9^ ^,^ ^13^. In the school environment, health is promoted through (among other things) the quality of the physical and social space; cultural and organizational environment; and teaching techniques^2^ ^,^ ^27^. The World Health Organization (WHO) published recommendations to improve the health of schoolchildren, educators, family members, and surrounding communities, stressing the importance of the school environment in building the health of future generations^9^ ^,^ ^13^.

Health promotion in school has a wide range, with repercussions in the communities and collective production of health-related knowledge^13^. Each school is integrated with the health services network, and, as most health determinants^3^, reflects different social, economic, and environmental conditions of its surroundings and their occupants. These different conditions are usually studied in terms of social inequalities, which, translated for the country’s internal geographic strata, are presented as regional inequalities^16^ ^,^ ^17^. Different possibilities of access to goods and services, different maintenance conditions and equipment supply of the schools may determine distinct conditions and diverging possibilities of guaranteeing the quality of the service provided.

According to Donabedian^6^, a guarantee of health quality is the combination of all actions taken to establish, promote, protect, and improve the quality of the care offered. He proposed a model to evaluate health services that involve the combined analysis of three dimensions: structure, process, and result. Structure refers to the general conditions in which the care is offered. Process means the activities that constitute the care. Result refers to the state of health, as well as satisfaction indicators. Elements of these dimensions can be found in existing environmental assessment instruments^12^ ^,^ ^21^ ^,^ ^25^, such as the WHO’s Global School-Based Student Health Survey (GSHS), including a specific module for environmental evaluation, answered by school representatives^20^ ^,^ ^23^ ^,^ ^26^. These models tend to be adapted for the experiences, needs, and characteristics of their regions of origin, or are excessively comprehensive^13^ ^,^ ^14^ ^,^ ^16^. Others are exact, being specific to certain topics^2^ ^,^ ^4^ ^,^ ^7^ ^,^ ^8^ ^,^ ^22^.

The National School-Based Health Survey (PENSE) is performed by the Ministries of Health and Education in partnership with the Brazilian Institute of Geography and Statistics (IBGE)^a^. In its 2012 edition, it included a module of interviews with school managers regarding the characteristics of the school environments to which ninth-year Brazilian students are exposed. The health promotion indicators investigated can be grouped into two dimensions: Structure and Process. This study analyses the data of PENSE-2012, aiming to identify aspects associated with the exposure of students to school environments that present better conditions for health promotion.

## METHODS

The PENSE was a cross-sectional study that interviewed schoolchildren in the ninth year (eighth grade) of public and private elementary schools. A managerial representative of each school answered a questionnaire regarding the school environment, developed specifically for this edition of PENSE. The PENSE-2012 sample represented Brazil and its five macroregions. For the sampling plan, 27 geographic strata were defined, corresponding to all the state capitals and the Distrito Federal. The remaining municipals were grouped into each of the five major geographic regions, forming five geographic strata. The sample of each stratum was allocated proportionally to the number of schools according to their administrative type (public or private). The study used separate sampling plans for the two groups of municipalities. We selected a sample of conglomerates in two stages for each of the 27 strata of capitals and the Distrito Federal, as follows: 1st stage – schools; 2nd stage – eligible classes in the selected schools (ninth year of elementary school). In the strata with minor municipalities, based on criteria of homogeneity and neighborhood, we formed groups of around 300 to 600 classes. We selected a sample of these groups in each region, and then selected the schools. Thus, for each of the five strata of the minor municipalities, conglomerates were sampled in three stages: 1st stage – group of municipalities; 2nd stage – schools; 3rd stage – eligible classes in the selected schools (ninth year of elementary school). In each stratum, the schools with less than 15 students in the target grade were excluded (less than 1% of the student total), as well as classes from the evening shift.

IBGE teams visited the classes selected in each school, and invited all students from these classes who were present on the day to participate in the study. The students answered the electronic questionnaire. The team interviewed a manager or representative of each school in the same visit. A total of 2,842 schools were selected, five of which declined to participate in the study. According to the schools visited, 84% of the students enrolled were present on the visiting days, adding up to 110,873 present schoolchildren. After 1,651 denials and 118 exclusions of questionnaires due to incomplete data (1.6% loss), the study included 109.104 interviews. The losses were within the expected limits for studies of this type. In addition, sample weights were calculated for the students who answered the survey, so as to represent the students enrolled in the ninth year of elementary school who regularly attended classes, according to the information obtained from the schools studied. The weight of each student was calculated by the product of the selection weights from each stage of the sampling process, corrected for the absence of the students who regularly attended classes, but who were not present on the date of interview.

In this analysis, we created a score with health promotion indicators in the school environment. The variable generated was called the health promotion in the school environment score (EPSAE). The items studied reflect conditions of structure and process in the schools; thus, the score is divided into two dimensions with these names. In this study, the items studied will not be considered result indicators. All factors were studied as dichotomous variables, with a score of 0 (zero) when the school representative indicated that the item was absent and 1 (one) when present. When the variable indicated harmful conditions, such as the school being in a violent area, the values were inverted, with 0 (zero) for the occurrence of the item and 1 (one) for its absence. The variables with inverted values are indicated by “negative” in the list below.

The Structure dimension encompassed the following items:

- School in a violent area – negative;
- Library available on site;
- Video room on site;
- Computer room on site;
- Internet access on site;
- Sports court on site;
- Usable locker rooms on site.

The Process dimension reflects the following factors:

- Physical activity in courtyard with instructor;
- Free sports after hours;
- School council that meets once a month or more;
- Smoking prohibited;
- Schoolchildren or teachers smoking in class – negative;
- Access to unhealthy foods in cafeteria or other – negative;
- Access to healthy foods in cafeteria or other point of sales, including sales at the gate or door of the school.

Unhealthy foods^4^ ^,^ ^10^ included: soda, sugary drinks, candy, ice cream, pastries, sweets, hamburgers, hot dogs (or similar), fried foods, industrialized snacks, cookies, and sweet or salty biscuits. Healthy foods were^4^ ^,^ ^10^: fruit, fruit salad, unsweetened natural juice, milk, and dairy beverages (except soy milk).

Seven items were in each dimension, distributed into four main topics – in other words, each topic was represented by one or, at most, two items. Under Structure, the following were portrayed: security in surroundings, support for educational activities (library and video room), technology resources (computer room and availability of internet), and infrastructure for sports. Security was included in this dimension because the construction option, which locates the school building in a certain region, is part of its structure, although the conditions that determine higher or low security levels around it do not depend on the school infrastructure. The Process dimension portrays actions to stimulate sports or physical activity, citizenship and participation, anti-smoking initiatives, and healthy eating. The EPSAE values were obtained considering that each item adds zero or one point, adding up to zero to seven points in each dimension. By a simple sum of the values, the total score varies from zero to 14 points.

The data underwent a descriptive analysis for the sample characteristics, according to demographic and socioeconomic data, and for the distribution of the schoolchildren according to the items assessed in the EPSAE for Brazil and each major region. Next, we measured the difference between the averages of the total score and that for each dimension in the scores of the schools interviewed in each region.

We performed ordinal regression for the data to determine the total EPSAE, understanding that a higher score indicates a better health promotion conditions in the environment to which the students are exposed. The independent variables were included in the model according to a hierarchical model, with the following distal variables: region of the country and whether the municipal is a state capital; intermediary variables: administrative type (public or private) and education of parents; proximal: sex, age, race, and paid job. This model was established so that the estimated chance of each individual being exposed to higher scores in the sum of indicators used in this study would be adjusted for the effect of the independent variables on the same level or above it. We established three analysis levels, as we recognize distinct levels for defining inequalities. The first level is geopolitical, expressed as characteristics of the regions and municipals, which affect the local population. We then see the profile of the teaching network of the school and the socioeconomic profile of the families of the interviewed schoolchildren, represented here by the parents’ education, as a more local set of inequality determinants. Lastly, the individual characteristics were considered. Those variables associated with the outcome, considering p ≤ 0.20 were maintained in the model. We considered associations with p < 0.05 as significant. The odds ratios for the outcome were estimated according to the independent variables and their insertion in the model. All analyses were preceded by sample weighting for the universe of schoolchildren in Brazil.

This study was approved by the Research Ethics Committee of the Ministry of Health (Item 192/2012, for Entry 16.805 of CONEP/MS on March 27, 2012).

## RESULTS

Table 1 shows the distribution of the schoolchildren according to demographic and socioeconomic variables. About two thirds of the students interviewed indicated that their parents had a basic level of education. Almost half were 15 years of age or older, which is greater than the typical age for the school year, and most of them reported not having a paid job.

**Table 1:** Distribution of schoolchildren, with confidence intervals of 95% (95%CI), according to demographic and socioeconomic data. PENSE, 2012. (N = 109,104)

Variable	Distribution (%)*	95%CI*
Region of country where school is located		
North	7.7	7.5–7.8
Northeast	25.7	25.2–26.1
Southeast	44.8	44.3–45.4
South	14.0	13.8–14.3
Midwest	7.8	7.7–8.0
School in state capital		
No	79.4	79.1–79.8
Yes	20.6	20.2–20.9
Educational network		
Public	83.5	83.1–83.9
Private	16.5	16.1–16.9
Maternal education		
Basic education complete or not	61.2	60.7–61.7
High school education complete or not	25.8	25.4–26.3
Higher education complete or not	13.0	12.7–13.3
Paternal education		
Basic education complete or not	67.8	67.4–68.3
High school education complete or not	21.1	20.6–21.5
Higher education complete or not	11.1	10.8–11.4
Age		
14 years or less	51.1	50.6–51.6
15 years or more	48.9	48.4–49.4
Self-declared Caucasian		
No	63.6	63.1–64.1
Yes	36.4	35.9–36.9
Has paid job		
No	88.1	87.7–88.4
Yes	11.9	11.9–12.3

^*^ Analysis preceded by sample weighting.

Table 2 shows the distribution of the schoolchildren according to the ESPAE items. The first seven variables indicate the school structure, translating important regional inequalities, such as higher exposure of the students in the Midwest to violent areas. The South and Northeast regions had the lowest percentages of students exposed to this condition.

**Table 2:** Distribution of schools in the country by macroregions according to the studied indicators of the health promotion in school environment score. PENSE, 2012. (N = 109,104)

Variable	Brazil		North Region		Northeast Region		Southeast Region		South Region		Midwest Region	
					
	%*	IC95%*	%*	IC95%*	%*	IC95%*	%*	IC95%*	%*	IC95%*	%*	IC95%*
School in violent area												
Rarely or never	58.7	58.2–59.2	54.5	53.6–55.3	66.9	66.1–67.7	55.2	54.3–56.1	66.9	66.0–67.9	41.5	40.6–42.3
Sometimes or always	41.3	40.8–41.8	45.6	44.7–46.4	33.1	32.3–33.9	44.8	43.9–45.7	33.1	32.4–34.0	58.5	57.7–59.4
Library?												
No	13.3	13.0–13.7	14.2	13.5–14.8	17.5	16.8–18.2	13.4	12.8–14.0	2.1	1.8–2.5	18.5	17.8–19.2
Yes	86.7	86.3–87.0	85.8	85.2–86.5	82.5	81.8–83.2	86.6	86.0–87.2	97.8	97.6–98.1	81.5	80.8–82.8
Video room?												
No	41.0	40.5–41.5	50.3	49.4–51.1	49.3	48.5–50.2	32.7	31.8–33.5	43.4	42.3–44.4	48.1	47.2–49.0
Yes	59.0	58.5–59.5	49.7	48.9–50.6	50.7	49.8–51.5	67.3	66.5–68.2	56.6	55.6–57.7	51.9	51.0–52.8
Computer room?												
No	11.8	11.5–12.1	20.3	19.5–21.0	17.9	17.2–18.6	7.3	6.8–7.7	8.2	7.6– 8.8	15.8	15.1–16.5
Yes	88.2	87.9–88.5	79.7	79.0–80.5	82.1	81.4–82.8	92.7	92.3–93.2	91.8	91.2–92.4	84.2	83.5–84.9
Internet access at school?												
No	24.8	24.3–25.2	36.7	36.0–37.7	31.0	30.2–31.8	22.4	21.7–23.2	10.1	9.5–10.8	30.9	30.0–31.7
Yes	75.2	74.8–75.7	63.1	62.3–64.0	69.0	68.2–69.8	77.6	76.8–78.3	89.8	89.2–90.5	69.1	68.3–70.0
Sports court?												
No	21.3	20.9–21.7	25.4	24.6–26.2	51.9	51.1–52.8	7.5	7.0– 7.9	3.8	3.5–4.2	27.4	26.6–28.2
Yes	78.7	79.3–79.1	74.6	73.8–75.4	48.1	47.2–48.9	92.5	92.1–93.0	96.2	95.8–96.5	72.6	71.8–73.4
Usable locker rooms?												
No	71.7	71.2–72.1	83.3	82.7–83.9	85.9	85.4–86.4	57.8	56.9–58.7	79.8	79.0–80.7	78.7	78.0–79.3
Yes	28.3	27.9–28.8	16.7	16.1–17.3	14.1	13.6–14.6	42.2	41.3–43.1	20.2	19.3–21.0	21.3	20.7–22.0
Physical activity in courtyard with instructor?												
No	47.5	47.0–48.0	49.6	48.8–50.5	54.7	53.8–55.5	45.1	44.2–46.0	44.1	43.1–45.2	42.5	41.7–43.4
Yes	52.5	52.0–53.0	50.4	49.5–51.2	45.3	44.5–46.2	55.0	54.0–55.8	55.9	54.8–56.9	57.5	56.6–58.3
Free after-hours sports?												
No	40.0	39.5–40.5	40.7	39.8–41.5	39.9	39.0–40.8	42.0	41.1–42.9	29.5	28.6–30.5	47.0	46.2–47.9
Yes	60.0	59.5–60.5	59.3	58.4–60.2	60.1	59.2–61.0	58.0	57.1–58.9	70.5	69.5–71.4	53.0	52.1–53.8
Council meets once a month or more?												
No	68.4	68.0–68.8	65.9	65.1–66.7	69.4	68.7–70.3	76.2	75.4–76.9	56.7	55.7–57.7	43.7	42.8–44.5
Yes	31.6	31.2–32.0	34.1	33.3–34.9	30.5	29.7–31.3	23.8	23.1–24.6	43.3	42.3–44.3	56.3	55.5–57.2
Smoking prohibited?												
No	11.0	10.7–11.3	12.0	11.4–12.6	16.9	16.2–17.5	8.8	8.3–9.3	6.7	6.2–7.2	11.3	10.7–11.9
Yes	89.0	88.7–89.3	88.0	87.4–88.6	83.1	82.5–83.8	91.2	90.1–91.7	93.3	92.8–93.8	88.7	88.1–89.3
Smoking inside school?												
No	70.7	70.3–71.1	75.1	74.4–75.8	66.8	66.0–67.7	74.6	73.8–75.4	71.1	70.2–72.0	55.8	54.9–56.7
Yes	29.3	28.9–29.7	24.9	24.2–25.6	33.2	32.3–34.0	25.4	24.6–26.2	28.9	28.0–29.8	44.2	43.3–45.1
Access to unhealthy foods?												
No	10.0	9.6–10.3	5.4	5.0–5.7	6.3	5.9–6.7	15.8	15.1–16.5	1.4	1.2–1.6	8.6	8.1–9.2
Yes	90.0	89.7–90.4	94.6	94.3–95.0	93.5	93.3–94.1	84.2	83.5–84.9	98.6	98.4–98.8	91.4	91.0–91.9
Access to healthy foods?												
No	53.4	52.3–53.9	49.0	48.1–50.0	63.1	62.3–63.9	48.1	47.2–49.0	57.3	56.2–58.3	48.8	48.0–49.7
Yes	46.6	46.1–47.2	51.0	50.1–51.9	36.9	36.1–37.7	51.9	51.0–52.8	42.7	41.7–43.8	51.2	50.3–52.0

^*^ Analysis preceded by sample weighting.

As for technological equipment, 13.3% of the Brazilian students attended schools that did not have libraries, while 11.8% did not have access to computer rooms, and 24.8% attended schools without Internet access. In the South, almost all students attended schools with libraries, while in the Midwest, this option was available for 81.5%. Access to video rooms in the schools varied from 49.7% in the North to 67.3% in the Southeast. The North region had the highest percentage of students without access to computer rooms or the Internet at school.

About four of five schools offered courts for sports, but only 28.3% had usable locker rooms. The Northeast had the lowest offer of sports courts, where about half of the schoolchildren did not have access to one, while the South had the highest offer.

The second group of seven variables in Table 2 shows Process indicators, which translate school actions and policies. While 90% of the interviewees in the country had access to unhealthy foods, and less than half of them had access to healthy foods. Most of the students in the South (98.6%) were exposed to unhealthy foods, while the Southeast had the lowest rate of this occurrence (84.2%). Healthy foods were absent in schools frequented by 63.1% of the students in the Northeast and 48.1% of those in the Southeast.

Almost half of the schoolchildren in the country had no access to physical activities in the courtyard with an instructor, and 40.0% went to schools that did not offer free sports after school hours. Physical activity stimulation is seldom referred to, but is not the source of regional inequalities. Service to students in schools with physical activity in the courtyard with an instructor varied little, and was lower in the Northeast and higher in the Midwest. For free sports offered after hours, the inequalities became more evident, with the South having the greatest proportion of students with this item and the Midwest, the lowest.

Around two thirds of the interviewees were at schools without an active School Council. The Southeast had the worst performance for active School Council, while the Midwest students attended schools with this item.

As for anti-smoking, 11.0% of Brazilian schoolchildren were still in schools that did not prohibit smoking, and 29.3% went to schools where the management was aware that students and teachers smoked inside the building. The report on anti-smoking rules presented the least inequalities among the regions. As for the perception of smoking in the school environment, the Midwest had the highest proportion of exposed students (44.2%) and the North, the least (24.9%).

The EPSAE average of the schoolchildren in the country was 8.35 points (SD = 2.18). Schools in the South and Southeast obtained the highest total scores, with averages at 8.98 points (SD = 1.79) in the South, and 8.84 points (SD = 1.98) in the Southeast. The lowest average score was for the Northeast, with 7.42 points (SD = 2.27). The difference between the averages was significant, mainly due to the differences in the Structure dimension, where the average scores of the South and Southeast are notable (Table 3).

**Table 3:** Health promotion in school environment (EPSAE) total scores and by dimensions attributed to interviewees, according to the evaluation of the school each frequented, in the country and by macroregion. PENSE, 2012. (N = 109,104)

Region	EPSAE^a^		Structure dimension^a^		Process dimension^a^	
		
	Mean	DP	Mean	DP	Mean	DP
Brazil	8.35	2.18	4.75	1.54	3.60	1.25
North	7.87^b^	2.17	4.24^b^	1.54	3.63^b^	1.21
Northeast	7.42^b^	2.27	4.13^b^	1.52	3.29^b^	1.39
Southeast	8.84^b^	1.98	5.14^b^	1.46	3.70^b^	1.19
South	8.98^b^	1.79	5.19^b^	1.14	3.78^b^	1.11
Midwest	7.93^b^	2.35	4.22^b^	1.74	3.71^b^	1.23

^a^ Analyses preceded by sample weighting.

^b^ p < 0.001 for difference between averages.

Table 4 shows that, among the conditions evaluated, after the adjusted analysis, it is more likely for a student to frequent a school with high EPSAE in the South (OR = 2.80; 95%CI 2.67–2.93) and Southeast (OR = 2.38; 95%CI 2.27–2.49) if the municipal is a state capital (OR = 1.97; 95%CI 1.90–2.03), if the mother (OR = 1.39; 95%CI 1.30–1.47) or father (OR = 1.29; 95%CI 1.21–1.37) have higher education, if the student is 15 years of age or older (OR = 1.20; 95%CI 1.16–1.24), if they have paid jobs (OR = 1.06; 95%CI 1.01–1.12) and, mainly if the school is private (OR = 4.52; 95%CI 4.25–4.81).

**Table 4:** Crude and adjusted odds ratios, estimated through ordinal regression, with respective confidence intervals of 95% for the health promotion in school environment score (EPSAE). PENSE, 2012. (N = 109,104)

Variable	Crude analysis*			Adjusted analysis*		
	
	OR	IC95%	p	OR	IC95%	p
Region of the Country			< 0.001			< 0.001
North	1			1		
Northeast	0.63	0.61–0.66		0.68	0.65–0.72	
Southeast	2.24	2.14–2.34		2.38	2.27–2.49	
South	2.43	2.32–2.54		2.80	2.67–2.93	
Midwest	1.06	1.02–1.12		1.02	0.97–1.06	
Municipal where located			< 0.001			< 0.001
Is not a capital	1			1		
Is state capital	1.80	1.74–1.86		1.97	1.90–2.03	
Educational network			< 0.001			< 0.001
Public	1			1		
Private	5.56	5.26–5.87		4.52	4.25–4.81	
Maternal education			< 0.001			< 0.001
Basic education complete or not	1			1		
High school education complete or not	1.48	1.42–1.54		1.10	1.05–1.15	
Higher education complete or not	2.97	2.81–3.13		1.39	1.30–1.47	
Paternal education			< 0.001			< 0.001
Basic education complete or not	1			1		
High school education complete or not	1.60	1.53–1.67		1.12	1.06–1.17	
Higher education complete or not	3.04	2.87–3.23		1.29	1.21–1.37	
Sex			< 0.001			0.521
Female	1			1		
Male	1.08	1.04–1.11		1.01	0.98–1.05	
Age			< 0.001			< 0.001
Up to 14 years	1			1		
15 years or more	1.55	1.50–1.61		1.20	1.16–1.24	
Self-declared Caucasian			< 0.001			0.367
No	1			1		
Yes	1.51	1.46–1.57		1.02	0.98–1.06	
Paid job			0.002			0.022
No	1			1		
Yes	0.93	0.88–0.97		1.06	1.01–1.12	

* Analysis preceded by sample weighting.

## DISCUSSION

This study presents a set of indicators available in PENSE-2012 that reflect conditions to which schoolchildren in the country and its five regions are exposed, and also maps inequalities in relation to this score. The registration of the conditions observed could stimulate relevant reflections in this field. Health promotion is undergoing a fifth wave in public health, where the maximization of the value of health and incentives to healthy behavior are prominent, promoting healthy choices and motivating the minimization of factors promoting unhealthy behavior^5^. School is a social space and potential health promotion environment, where children and adolescents spend time over many years during the first decades of life. They are institutions of reference for entire communities, and can influence and contribute to health promotion efforts in their surrounding areas. Health in school is a combination of health education and health protection actions^1^. This study presents important inequalities regarding the conditions to which our children and teenagers are exposed in the schools they frequent, considering both the country’s regions and the characteristics of the municipals of the schools for the analyses with variables of a more local level or even regarding individual characteristics of the students.

The low number of indicators available for composing the EPSAE is a limitation of this study. More indicators, encompassing a wider range of topics that are already part of the health promotion guidelines in schools, would lead to an even more exact diagnosis of the conditions to which Brazilian schoolchildren are exposed. Other indicators, which were not considered in this analysis because they were not investigated in the questionnaire applied to school managers in PENSE-2012, aim to identify another profile of inequalities; for example, attention to sanitary conditions, data on the social environment, topics such as STD, care in the parent-school relationship, and healthcare actions offered directly to the students^11^ ^,^ ^14^ ^,^ ^24^. These topics could generate indicators for any of the dimensions studied, or for results. The study also excluded result indicators by opting to consolidate the analyses of Structure and Process indicators, since the use of result indicators would require specific steps and in-depth study of each of the topics defined as results. Another limitation of the study was that the questionnaire applied to the school managers was not validated, so the information could be biased. However, the large quantity of schools visited and the standardization of the questionnaire and its application may have minimized this risk.

The indicators examined considered fundamental items for health promotion in schools^13^. In Brazil, the National Health Promotion Policy highlighted the support to schools that promote health mainly for, among other things: healthy eating, physical activities, and tobacco-free environment^b^. The Health in Schools program strengthened the fight against the vulnerabilities, maintaining these priorities and highlighting others^c^. With indicators for structure and process, the score seems well dimensioned and considers these priorities. The data obtained show important inequalities, considering both the examination of the distribution of schoolchildren according to EPSAE items, in Table 2, and the revision of the average scores per region, in Table 3. The South and Southwest regions of the country tend to have better scores and a greater proportion of students in schools that include the indicators evaluated. Inequality is one of the relevant concerns regarding access to health services or technologies in education^3^ ^,^ ^19^. The first movement towards reverting this scenario is to highlight this reality^17^.

Inequality in the Structure dimension seems diluted among the indicators, with no notable items in particular. Inequality promotes a sort of selective technological illiteracy^17^, as some schoolchildren face less equipped environments, which tends to justify and maintain socioeconomic inequalities. This interferes directly, for example, in access to job markets. The inequalities among regions seem to follow those observed for the location of the schools in state capitals, for the education of the parents, and for the private schooling network. The composition of the differences found in the EPSAE suggests a case of inequality determined by conditions that do not depend on the will of the individuals. It may be that the students do not opt to attend schools with less expressive scores. The difference between the scores for those enrolled in private schools is even more evident than the disparities between regions. If education, offered as a public service, is conceded to a private institution, why are there such expressive differences between schools? A study performed in 25 countries^18^ suggests that health inequalities are associated not only with economic and financial limitations, but with a fragile civil society. This reinforces the belief in the structural model for understanding health inequalities^15^; however, this scenario can be interpreted from the perspective of the fragility of civil society, indicating a regime of tolerance with differences that produce inequalities and limit the sense of democracy. The findings of this study do not satisfy the principle of equity. The students with better health promotion conditions at school have mothers and fathers with higher education, which can indicate higher income, and study in the capitals and in regions with greater gross domestic product.

The independent analysis of dimension, however, does not reproduce the same panorama. The distribution of the schoolchildren according to the process is similar among the regions (Table 2). The scores of the regions (Table 3), with averages very close to half of the possible points, reflect great fragility in the country and in all regions in terms of the actions and policies for health promotion in schools. The average score for Process is always lower than the Structure dimension. There is heavier investment in equipment than in the care and development of skills and abilities. The visibility of resources and equipment, guaranteed by better economic-financial conditions, may contribute to the blurred perception of the process-related weaknesses.

When the indicators are reviewed separately, the data obtained indicate that Brazil still cared very poorly for the student food offering in 2012. Tobacco use seems to have been handled more effectively than the nutrition. However, actions to promote physical activity do not accompany the offer of structural conditions, such as appropriate sports courts. The development of health policies in school means the formulation of clearly defined guidelines that can guide actions and allocation of resources in health promotion areas^13^. The data presented here do not translate the idea of health promotion in schools, and difficulties including the regional inequalities are part of the historic process of the effort for health promotion in schools^9^.

PENSE contributes expressively to the accumulation of information, and its serial nature will allow comparisons that can indicate the direction in which the country is moving. The integration of health and education sectors in new arrangements, in a participative fashion, as an attempt to break with prescriptive, unarticulated, and focalized tradition, is already among the latest guidelines of the Health in School Program. The inequalities found here can be reverted. The indicators available do not cover the totality of the health priorities, but the inequalities can also reflect the performance of the regions regarding the number of schoolchildren exposed to the negative (undesirable) category of each indicator. The Southeast region, for example, did not have the worst performance in any of the indicators, and was the best in five of the 14 indicators available. The South offered the best conditions in six indicators, but had the worst score for the item regarding access to unhealthy foods. The North and Northeast regions scored best in only one indicator. The Northeast had the greatest exposure of students to negative conditions, with the worst performance in six items: sports court and locker room in usable conditions; physical activity in courtyard with instructor; regularly active School Council; prohibition of smoking in school environment; and access to healthy foods. The North scored the worst in supporting technology resources (video room, computer room, and access to internet at the school). The Midwest performed best in two indicators, while having the most exposure of students to violent areas, without libraries or the free offer of sports after school hours. In addition, the Midwest presented a higher number of schoolchildren in schools where occurrences of smoking on site were registered (Table 2).

Our findings allow us to conclude that important inequalities persist regarding health promotion indicators in schools in Brazil, highlighting the differences between the regions of the country and between public and private schools. These inequalities are more evident among structural indicators, and show the need for more significant investment in Process indicators for the country as a whole. In this regard, fewer inequalities appear, but the scores obtained can be considered low. Future studies will be able to explore the relationship between these indicators and result indicators.

## Footnotes

a Instituto Brasileiro de Geografia e Estatística. Pesquisa Nacional de Saúde do Escolar 2012. Rio de Janeiro: IBGE; 2012 [cited 2017 Jan 18]. Available from: http://www.ibge.gov.br/home/estatistica/populacao/pense/2012/

b Ministério da Saúde (BR), Secretaria de Vigilância em Saúde, Secretaria de Atenção à Saúde. Política Nacional de Promoção da Saúde. Brasília (DF); 2006. (Série B. Textos Básicos de Saúde).

c Ministério da Saúde (BR); Ministério da Educação (BR). Programa Saúde na Escola. Brasília (DF): Ministério da Saúde; 2010 [cited 2012 Jun 25]. Available from: http://dab.saude.gov.br/programa_saude_na_escola.php
